# Hydrophobic Residues near the Bilin Chromophore-Binding Pocket Modulate Spectral Tuning of Insert-Cys Subfamily Cyanobacteriochromes

**DOI:** 10.1038/srep40576

**Published:** 2017-01-17

**Authors:** Sung Mi Cho, Sae Chae Jeoung, Ji-Young Song, Ji-Joon Song, Youn-Il Park

**Affiliations:** 1 Department of Biological Sciences, Chungnam National University, Daejeon 34134 Korea; 2 Center for Advanced Measurement and Instrumentation, Korea Research Institute of Standards and Science, Daejeon 34113 Korea; 3 Department of Biological Sciences, Korea Advanced Institute of Science and Technology, Daejeon 34141 Korea

## Abstract

Cyanobacteriochromes (CBCRs) are a subfamily of phytochrome photoreceptors found exclusively in photosynthetic cyanobacteria. Four CBCRs containing a second Cys in the insert region (insert-Cys) have been identified from the nonheterocystous cyanobacterium *Microcoleus* B353 (Mbr3854g4 and Mbl3738g2) and the nitrogen fixing, heterocystous cyanobacterium *Nostoc punctiforme* (NpF2164g3 and NpR1597g2). These insert-Cys CBCRs can sense light in the near-UV to orange range, but key residues responsible for tuning their colour sensitivity have not been reported. In the present study, near-UV/Green (UG) photosensors Mbr3854g4 (UG1) and Mbl3738g2 (UG2) were chosen for further spectroscopic analysis of their spectral sensitivity and tuning. Consistent with most dual-Cys CBCRs, both UGs formed a second thioether linkage to the phycocyanobilin (PCB) chromophore via the insert-Cys. This bond is subject to breakage and relinkage during forward and reverse photoconversions. Variations in residues equivalent to Phe that are in close contact with the PCB chromophore D-ring in canonical red/green CBCRs are responsible for tuning the light absorption peaks of both dark and photoproducts. This is the first time these key residues that govern light absorption in insert-Cys family CBCRs have been identified and characterised.

Cyanobacteriochromes (CBCRs) with single or multiple bilin-binding c**G**MP-specific phosphodiesterase, **A**denylyl cyclase and **F**hlA (GAF) domains are cyanobacterial photosensory proteins that are distantly related to phytochromes (Phys). These proteins reversibly interconvert between dark-stable and photoproduct states upon photoisomerisation of their linear tetrapyrrole (bilin) chromophores^1^. Some Phys and CBCRs are involved in regulating light acclimation processes such as phototaxis^2^,^3^,^4^,^5^,^6^,^7^,^8^, chromatic acclimation^9^,^10^,^11^,^12^,^13^ and light-dependent cell aggregation^14^,^15^. Cyanobacterial Phys and CBCRs mostly use phycocyanobilin (PCB) as a chromophore precursor, and photoisomerisation of the 15,16-double bond of the bilin chromophore is the primary photochemical reaction during photoconversion (Supplementary Fig. S1). Unlike most cyanobacterial Phys that undergo photoconversion between a red-absorbing dark state and a far-red-absorbing photoproduct, photostates of CBCRs cover near-UV^5^,^16^,^17^, violet^16^,^17^,^18^, blue^15^,^16^,^17^,^19^,^20^,^21^,^22^,^23^, teal^20^,^22^,^23^, green1^15^,^17^,^19^,^23^,^24^,^25^,^26^, orange^16^,^17^,^20^,^23^,^26^, red^10^,^21^,^23^,^25^,^26^ and far-red^27^ wavelengths.

The diverse photocycles observed in most single-Cys CBCRs are due to spectral tuning mechanisms such as protochromism^12^, hydration^28^, PCB/phycoviolobilin (PVB) isomerisation^29^ and trapped-twist^30^, all of which influence the conformation and configuration of the bound bilin chromophore. In the trapped-twist model, Phe residues conserved in red/green CBCRs (Supplementary Fig. S1) constrain chromophore movement after the primary photoisomerisation event, which effectively traps the D-ring in an unconjugated state. Unlike these single-Cys GAFs, dual-Cys CBCR GAFs use a second Cys residue in the highly conserved Asp-Xaa-Cys-Phe (DXCF) motif, the poorly conserved CXXR/K motif in the insertion loop (insert-Cys) or, in AM1_1186g2, a distinctive Cys residue located at the helix α3 region of AnPixJg2 to form a second thioether linkage to the C10 atom of the bilin chromophore (Supplementary Fig. S1). Except in the insert-Cys CBCR UV/blue photocycle protein 1 (UB1) NpR1597g2, these second thioether covalent bonds are not stable, and these labile bonds undergo reversible formation and breakage during photoconversion, resulting in spectral tuning with wavelength optima from near-UV to red^5^,^16^,^17^,^21^,^31^,^32^,^33^ (Supplementary Fig. S1). In some dual-Cys-containing DXCF-type GAFs, the PCB chromophore isomerises into the PVB form, which results in blue to green (or teal, yellow or orange) spectral tuning^5^,^17^,^20^,^32^.

Recently, two near-UV(U)/green(G) CBCRs, UG1 Mbr3854g4 and UG2 Mbl3738g2 (Supplementary Fig. S2), were identified by searching for bilin photoreceptors in the genome of the filamentous nonheterocystous cyanobacterium *Microcoleus* B353^17^. Recombinant CBCRs UG1 and UG2 coexpressed with a PCB biosynthetic operon in *E. coli* (Supplementary Fig. S2) displayed a near-UV-absorbing dark ground state, with absorption maxima at 384 and 381 nm. These proteins are reversibly photoconvertible into green-absorbing photoproducts with absorption maxima at 560 and 556 nm, respectively. Thermal (dark) reversion of the photoproducts to their short wavelength-absorbing dark states was not observed for either of these UGs. Light-minus-dark difference spectra of acid-denatured UG1 and 2 (Supplementary Fig. S2) revealed that both UGs assembled with PCB in the 15*Z* configuration in the dark, and both were photoconverted to the 15*E* configuration.

Multiple sequence alignment of these GAFs against previously reported insert-Cys GAFs VO1 (NpF2164g3) and UB1 (NpR1597g2)^16^,^17^ (Fig. 1) revealed the presence of the putative insert-Cys (position IV). Interestingly, residues corresponding to Phe in the β1 notch (position I) of the distinctive red(R)/green(G) CBCR NpR3784 and its homologs^34^ and the β2 (position III) and α4 helix (position X) (Supplementary Fig. S1) that are involved in the trapped-twist mechanism of red/green and Teal-DXCF photocycles^30^ are only loosely conserved in insert-Cys CBCRs. Intriguingly, the insertion loop and the DXCF and CH motifs of VO1 from *N. punctiforme* are subject to light-dependent structural changes that include conversion from a random-coil structure in the dark state to a stable α-helical structure in the photoproduct, whereas the α-helical and β-sheet structure surrounding the D-ring of the chromophore remains almost unchanged during the dark-to-light transition^35^ (Fig. 1). Thus, it is probable that both specific and general features observed in the photocycles of insert-Cys GAFs are related to variation in loosely conserved amino acids in the insertion loop and other parts of the bilin-binding pocket, including residues in position I, III and X.

In the present work, we investigated the spectral properties (wavelength optima) in the photocycles of insert-Cys CBCRs using a series of recombinant UG variants produced in *E. coli* engineered for coproduction of the PCB bilin chromophore^36^. Application of thiol modifying reagents such as dithiothreitol (DTT), β-mercaptoethanol (βME) and iodoacetoamide (IAM) resulted in red-shifted intermediates relative to the dark or light states present during the U/G photoconversion cycle. We also identified variations in Phe equivalents at the β2 (position III) and α4 helix (position X) surrounding the PCB D-ring that are responsible for spectral tuning in UGs. Our results strongly indicate that the geometry of PCB is constrained by residues equivalent to key Phe residues (positions III and X), and this constraint is responsible for spectral tuning in insert-Cys CBCRs. Our findings might help to identify similar CBCRs in other species, and could assist engineering of the spectral properties of these light-sensitive proteins.

## Results

### CBCR UG1 and UG2 from *Microcoleus* B353 are novel insert-Cys GAFs with UG photocycles

**Figure 1:**
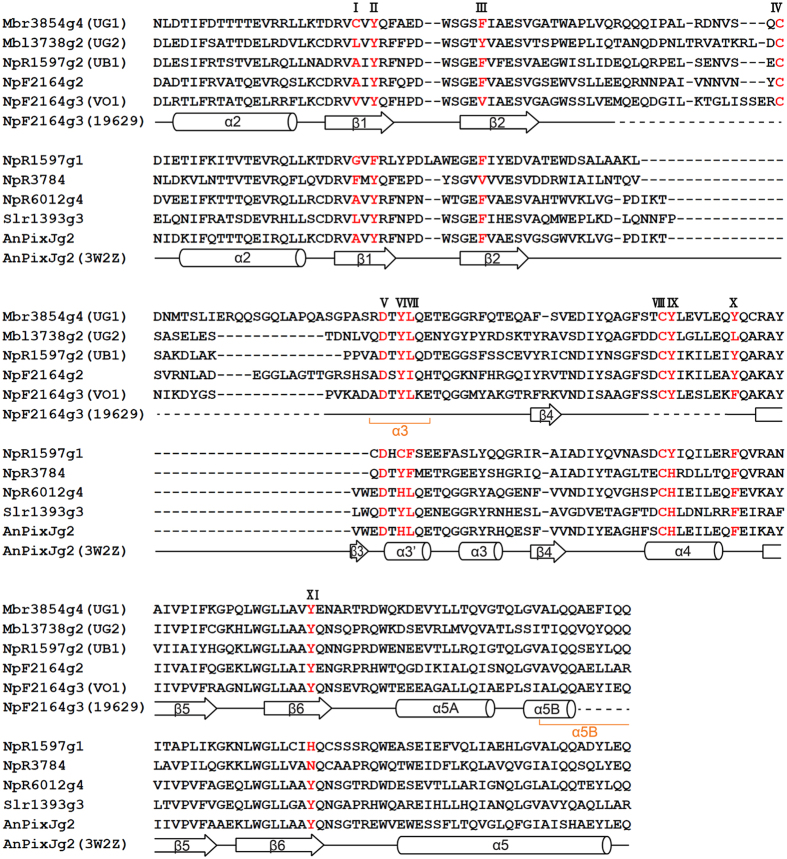
Multiple protein sequence alignment and secondary structures of insert-Cys GAFs. Insert-Cys CBCR GAF subclasses with violet/orange (NpF2164g3), near-UV/blue (NpF2164g2 and NpR1597g2) and near-UV/green (Mbr3854g4 and Mbl3738g2) photocycles are clustered. Red/green GAFs NpR3784, NpR6012g4, Slr1393g3, and AnPixJg2 and Teal-DXCF NpR1597g1 are included for comparison. Secondary structural elements of NpF2164g3^35^ (BMRB no. 19150) and AnPixJg2^38^ are illustrated for comparison. Key residues of insert-Cys CBCRs discussed in the main text are indicated with Roman numerals. β1 notch (I), β2 Phe (III), DXCF motif Leu (VII) and helix Phe (X) are associated with the photoproduct blue-shift of red/green CBCRs^20^,^31^,^34^.

**Figure 2:**
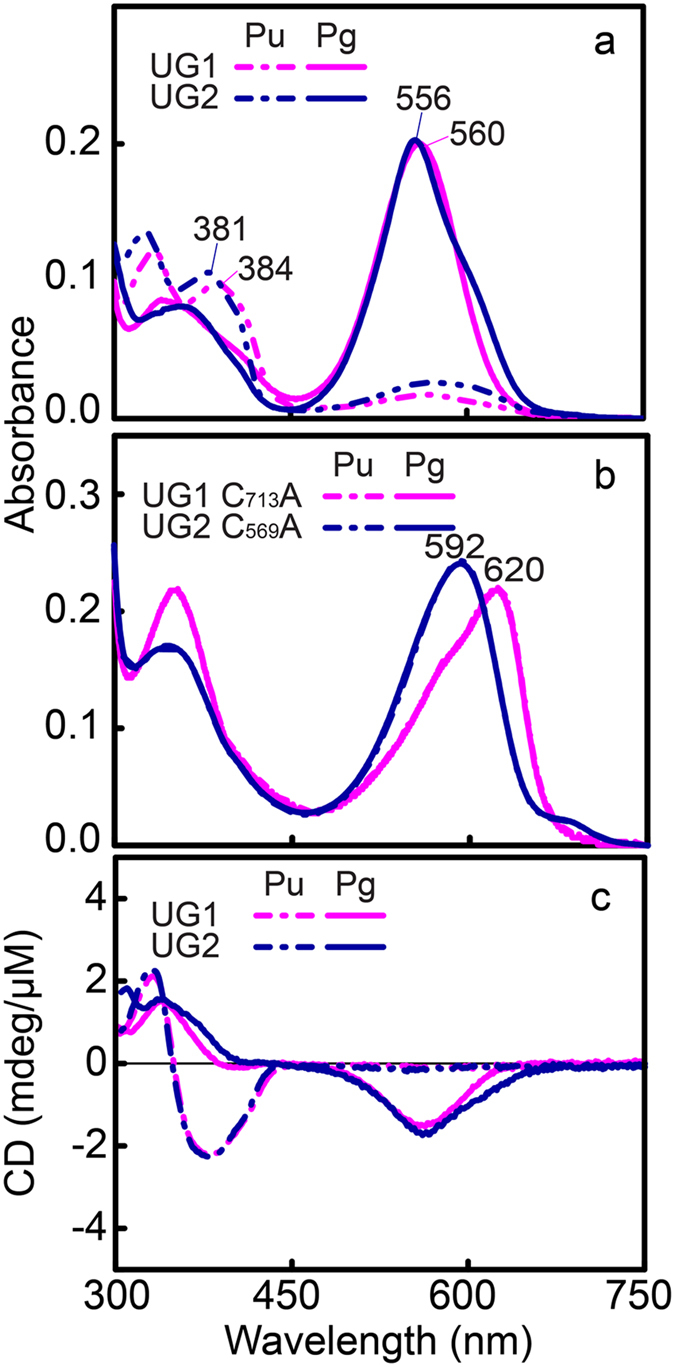
Newly identified insert-Cys CBCRs Mbr3854g4 (UG1) and Mbl3738g2 (UG2) from *Microcoleus* B353 are near-UV/green photosensors. (**a**) Absorption spectra of wild-type UG1 and UG2. (**b**) Absorption spectra of insert-Cys variants UG1 C_713_A and UG2 C_569_A, showing red-shifts in their absorption peaks. (**c**) Circular dichroism spectra of wild-type UG1 and UG2.

UG1 and 2 include the insert-Cys feature, which refers to C_713_ and C_569_ within the inserted loops (Fig. 1). These residues are equivalent to C_298_ of UB1 and C_546_ of VO1 that form a second covalent linkage to the C10 carbon atom of the PCB chromophore^17^. As expected, variants of UG1 and 2 in which these Cys residues are substituted with Ala (C_713_A and C_569_A) exhibited normal PCB chromophore-binding activities, as revealed by Zn^2+^-dependent fluorescence (Supplementary Fig. S3). However, unlike wild-type UGs (Fig. 2a), dark-isolated UG1 C_713_A and UG2 C_569_A variants absorbed mostly red (620 nm) and orange (592 nm) light, respectively, and irradiation with either violet (380 nm) or red (640 nm) light produced no further significant changes in the absorption spectra (Fig. 2b, Supplementary Table S1). Similar red-shifts of differing extent relative to the denatured 15*E* PCB in acidic solution (*~*590 nm)^37^ were also observed in C_546_A and C_298_A variants of the respective orange and blue light-absorbing VO1 and UB1^16^. This implies that the twisted geometries of the PCB chromophore-binding pockets are variable in these three insert-Cys CBCRs, leading to shortening of the π-conjugation of both dark states and photostates to different degrees.

During photocycles of Phys and CBCRs, circular dichroism (CD) signals may be inverted, as occurs in Cph1 and plant Phys^1^, or remain unchanged, as occurs in DXCF CBCRs^19^. Consistent with insert-Cys CBCRs UB1 and VO1^16^, CD signals from both UGs were not altered during photoconversion (Fig. 2c). CD peak wavelengths were well correlated with those of absorption spectra from respective 15*Z* and 15*E* states with comparable signal strength, indicating that the facial disposition of the D-ring during the photocycle is conserved similarly in insert-Cys CBCR subfamily members and other DXCF CBCRs^20^.

### Photoproduct intermediates are transiently produced during forward and reverse photoconversions

**Figure 3:**
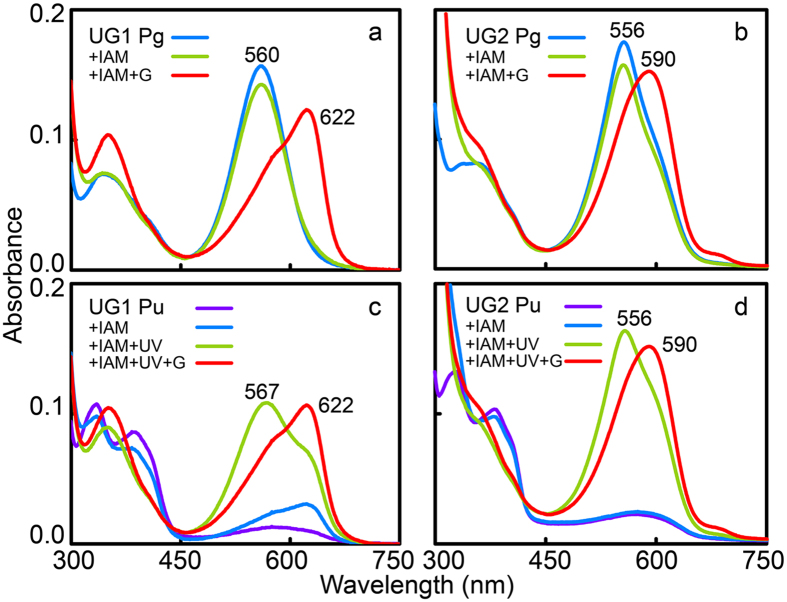
Differential susceptibility of the second thioether linkage of UG1 and UG2 to iodoacetamide (IAM) treatment. (**a,b**) Reverse (15*E* to 15*Z*) and (**c,d**) forward (15*Z* to 15*E*) photoconversion in UG1 (**a,c**) and UG2 (**b,d**) were examined after treatment with 50 mM IAM. Photoproteins were converted to the 15*E* state (blue), followed by reaction with IAM in darkness to yield 15*E* chemical products (green). The 15*E* chemical products were then illuminated when appropriate to generate 15Z photoproducts (red). The UG1 dark state is sensitive to IAM treatment, whilst that of UG2 is resistant.

The photo-labile nature of the second Cys-mediated thioether bond in insert-Cys CBCR VO1 during forward photoconversion was illustrated in the presence of the thiol inhibitor IAM that binds to Cys residues^16^,^20^. This photo-lability of the second covalent linkage was also observed in both UGs. Both green-absorbing UG1 and 2 treated with IAM failed to convert into near-UV-absorbing forms, but rather formed red (Pr, 622 nm)- or orange (Po, 590 nm)-absorbing photoproducts (Fig. 3a,b). Photoconversion from Pu to Pg forms was hardly affected by the IAM treatment, but subsequent green illumination trapped Pr and Po photoproducts (Fig. 3c,d). Interestingly, the IAM-treated Pu form of UG1 exhibited an apparent increase in red-absorbing species (2.99-fold increase in absorbance at 622 nm), unlike the IAM-treated Pu form of UG2 (Fig. 3c *vs.* d), suggesting that the accessibility of the chromophore pocket for IAM entry is slightly different in the dark states of these UGs. Alternatively, this partial conversion of Pr to Pu after IAM treatment could be due to modification of a small amount of free Cys, as observed previously with AM1_1186g2^21^ and Tlr1999^22^.

**Figure 4:**
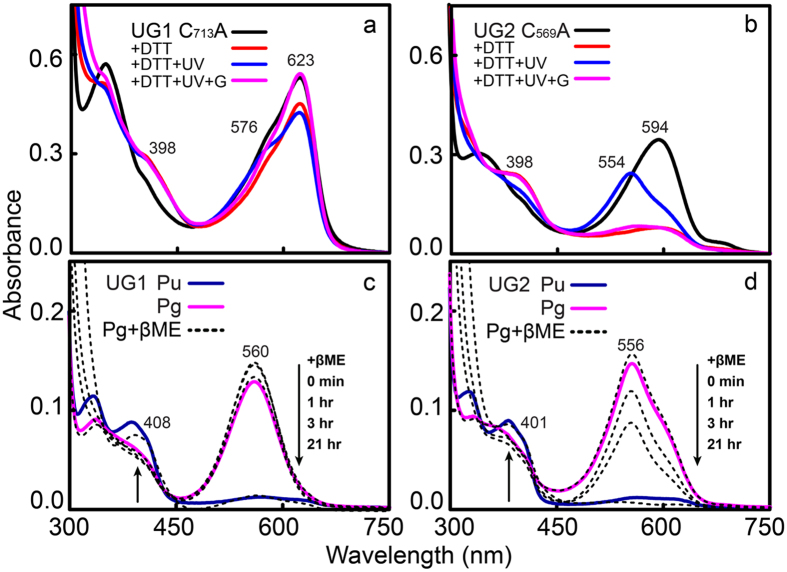
The thiol reagents dithiothreitol (DTT) and β-mercaptoethanol (βME) restore the second thiol linkage in the dark. (**a,b**) DTT treatment restores the photocycle of insert-Cys mutants UG1 C_713_A (**a**) and UG2 C_569_A (**b**). Both mutants were treated with 50 mM DTT (red) in the ground state, irradiated with near-UV light (blue), then illuminated with green light (pink). Unlike the partial restoration observed in UG1 C_713_A, full recovery of the photocycle was observed in the UG2 C_569_A mutant following addition of DTT. Note that red-shifted, violet-absorbing products (15Z, 397−420 nm) were generated, rather than near-UV-absorbing species (15Z). (**c,d**) βME treatment generates violet-absorbing photostates. Green light-absorbing wild-type UGs (15*E* Pg) treated with 1% βME in the dark slowly reverted into violet-absorbing forms with peaks at 408 nm for UG1 and 401 nm for UG2.

Restoration of the second thioether linkage via the insert-Cys residue during the reverse reaction was evident from the analysis of the effects of DTT on the insert-Cys variants of both UGs (Fig. 4). The thiol-containing reagent DTT acts as a second Cys and converts 15*E*-single-Cys to 15*E*-dual-Cys CBCR species, even in the dark^22^. As shown in Fig. 4a and b, DTT treatment transformed the dark-adapted, red (620 nm)- or orange (592 nm)-absorbing C_713_A UG1 and C_569_A UG2 variants (15*Z* PCB, thiol-free) partially or almost fully into the violet-absorbing ground state (15*Z* PCB, thiol-ligated by DTT, ~398 nm). Furthermore, DTT-treated UGs exhibited partial or full U/G photocycles. For instance, photoconversion of C_713_A UG1 in the presence of DTT was marginal relative to C_569_A UG2, consistent with previous studies on UB1 and VO1^16^. By contrast, UV-A illumination of DTT-treated C_569_A UG2 resulted in the formation of a green-absorbing photoproduct (15*E*, 554 nm), which in turn converted into the violet-absorbing (15*Z*, 403 nm) form (Supplementary Fig. S4). These results demonstrate that insert-Cys residues act to disrupt conjugation in the chromophore by forming a thiol linkage.

The observation of trapped photoproducts during reverse conversions in the presence of IAM implies the presence of photoproduct intermediates in which bound PCBs are in the 15*E* isomeric form, and with the second thioether remaining intact, as suggested previously for VO1^16^. We therefore searched for these intermediate species using DTT. In contrast to our expectations, DTT had little effect on the absorption spectra of the 15*E* PCB of either UG1 or 2 (data not shown). However, when βME, which is smaller than DTT, was added to wild-type green-absorbing UGs in the dark, the absorbance following green illumination slowly disappeared, whilst red-shifted violet absorption peaks (408 nm for UG1 and 401 nm for UG2) increased significantly, although the progression of this process differed in the two UGs (Fig. 4c,d). These results indicate that the violet-absorbing form (Pv, 15*E* PCB, thiol-ligated) was transiently produced during forward photocycles.

### Both the insertion loop and conserved Asp and Tyr residues in the DXCF motif are required for efficient photoconversion

**Figure 5:**
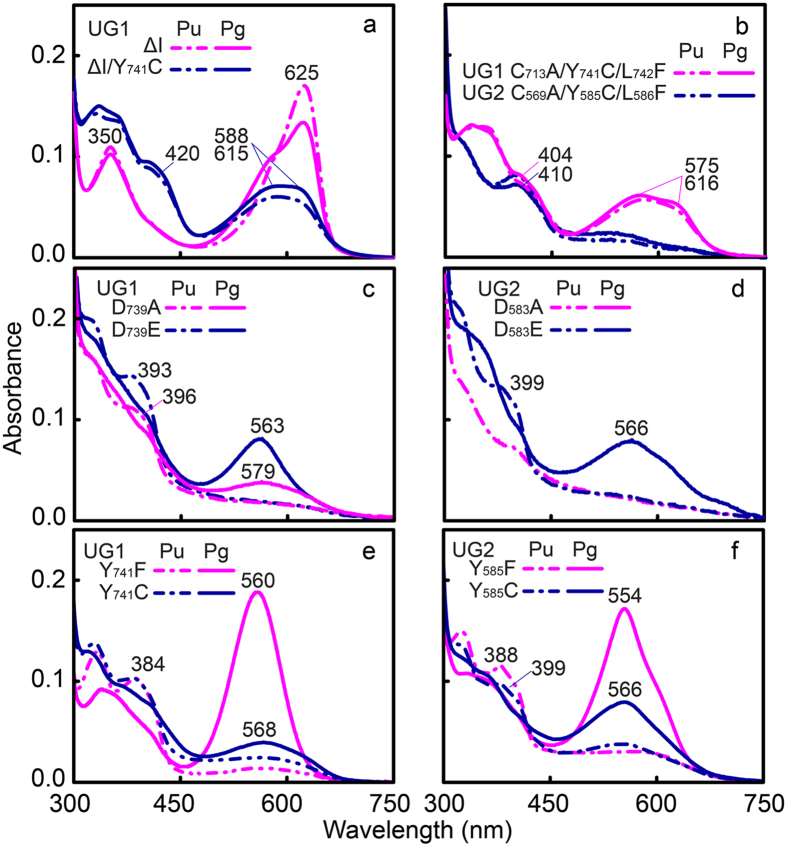
The insert-Cys-containing loop and the DXCF motif of insert-Cys CBCRs are required for photoisomerisation. (**a**) Absorption spectra of insertion loop deletion variants with (ΔI Y_741_C) or without (ΔI) additional substitution of Tyr with Cys in the DXCF motif of UG1. UG1 ΔI variants display inefficient red/green photocycles. Introduction of the second Cys in the DXCF motif is able to disrupt conjugation in the chromophore, but photoconversion is inefficient. (**b**) Absorption spectra of triple variants of UG1 (C_713_A Y_741_C L_742_F) and UG2 (C_569_A Y_585_C L_586_F). Mimicking the DXCF motif of CBCRs by introducing the appropriate mutations in the insert region results in inefficient photocycles, and dark and photostates with broad absorption peaks. (**c–f**) Absorption spectra of Asp variants of UG1 (D_739_A and D_739_E) (**c**) and UG2 (D_583_A and D_583_E) (**d**) and Tyr variants of UG1 (Y_741_F and Y_741_C) (**e**) and UG2 (Y_585_F and Y_585_C) (**f**). Spectra were recorded for the dark state, or after near-UV light irradiation. Asp and Tyr variants commonly displayed reduced photoconversion efficiencies.

In insert-Cys-type GAFs, the type and number of amino acid residues in the insertion loop vary between subfamily members (Fig. 1), suggesting that this region is not critical for conserved functions other than providing the second thioether linkage to the bilin chromophore. However, contrary to our expectations, this region was required for efficient photoconversion. As shown in Fig. 5a, when the insertion loop was deleted by mutation (ΔI), normal chromophorylation was observed (Supplementary Fig. S3), but the efficiency of photoconversion between red- and green-absorbing photoproducts was significantly reduced (Supplementary Table S1). Even replacement of the Tyr residue of the DXCF motif with Cys (ΔI Y_741_C) to mimic the canonical DXCF motif failed to recover the photoconversion efficiency. The triple variant C_713_A Y_741_C L_742_F UG1, in which DTYL was replaced by the canonical DXCF motif present in dual-Cys CBCRs (the number of amino acids in the insert loop was unchanged), also exhibited an inefficient photoconversion comparable to the ΔI Y_741_C variants (Supplementary Table S1). Consistently, the C_569_A Y_585_C L_586_F UG2 variant was also unable to complete U/G photocycles, although the absorption spectra were more similar to those of dark-adapted Pu forms (Fig. 5b). Thus, the insert-Cys residue in the insert loop is required for efficient photoconversion.

Multiple sequence alignment of UGs revealed conserved Asp (position V) and Tyr (position VI) residues in the DXCF motif that are also present in other Phys and CBCRs (Fig. 1). In VO1, this helical DXCF motif-containing region undergoes folding and unfolding during photocycles^35^; hence residues D_739_/Y_741_ and D_583_/Y_585_, equivalent to Asp and Tyr in UG1 and UG2, are expected to play a unique role in insert-Cys CBCRs. Our results showed that all Asp and Tyr variants of UG1 and 2 were capable of binding PCB (Supplementary Fig. S3), but the photoconversion efficiency was again lower in these mutants, albeit to varying degrees (Fig. 5, Supplementary Table S1). Substitution of D_739_ with Glu in UG1 (D_739_E, Fig. 5c) and D_583_ in UG2 (D_583_E, Fig. 5d) was less severe than substitution with Ala (UG1 D_739_A and UG2 D_583_A), suggesting that a negative charge is essential for establishing the green light-absorbing state. Tyr_741_ in UG1 (Fig. 5e) and Tyr_585_ in UG2 (Fig. 5f) were also critical for photochromicity. Substitution of Tyr_741_ with Phe in UG1 (Y_741_F and UG2 Y_585_F) maintained almost 90% of the photoconversion efficiency, whereas the photoconversion efficiency of Cys variants (Y_741_C and Y_585_C) of UG1 and UG2 was severely decreased (12% for UG1 and 20% for UG2, Supplementary Table S1). CD spectra for Asp and Tyr variants of both UG1 and UG2 were consistent with these findings: CD signals from the 15*E* states at longer wavelength were significantly decreased or completely lost, whilst shorter wavelength signals were reduced compared with those of wild-type UGs (Fig. 2c, Supplementary Fig. S5). Thus, Asp and Tyr in the DXCF motif appear to be important for efficient primary photoconversion.

### Colour tuning of both dark and photoproduct states is dependent on Phe equivalents in the red/green CBCR AnPixJg2 GAF

**Figure 6:**
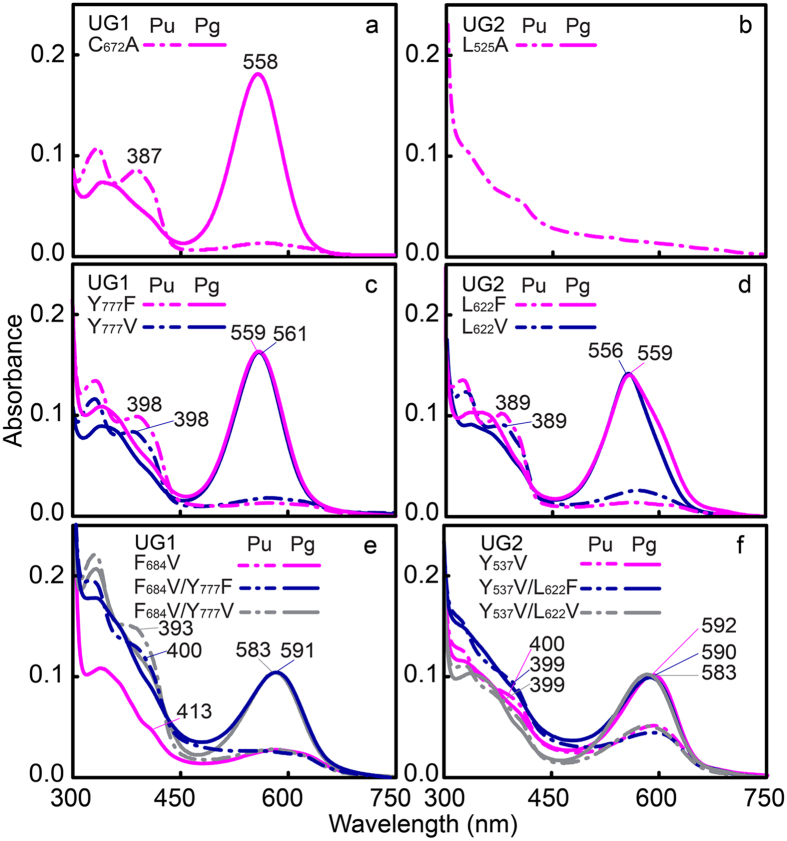
Hydrophobic residues in helix4 and β2 elements govern spectral tuning in the dark state and in photoproducts. (**a,b**) Absorption spectra of β1 notch equivalent variants C_672_A UG1 (**a**) and L_525_A UG2 (**b**). (**c,d**) Absorption spectra of helix α4 Phe equivalent variants Y_777_F (or V) of UG1 (**c**) and L_622_F (or V) of UG2 (**d**). Spectra are indistinguishable from respective wild-type UGs. Dark states display a red-shift to violet light absorption, whilst photostates are unaffected by the mutations. (**e,f**) Absorption spectra of β2 Phe equivalent single variant F_684_V UG1 (**e**) and Y_537_V UG2 (**f**) and additional substitution of helix α4 Phe equivalents F_684_V Y_777_F (or V) in UG1 (**e**) and Y_537_V L_622_F (or V) in UG2 (**f**).

We next analysed C_672_, F_684_ and Y_777_ of UG1 and L_525_, Y_537_ and L_622_ of UG2, which are located at the β1 notch (position I) in the NpR3784 group, and at β2 (position III) and helix-4 (position X) in the red/green CBCR AnPixJg2 (PDB ID: 3W2Z) (Fig. 1, Supplementary Fig. S1), respectively. These residues are implicated in spectral tuning in distinctive R/G CBCR NpR3784 homologs and canonical R/G CBCRs such as NpR6012g4 and Teal-DXCF by trapping the chromophore in a twisted geometry^30^,^34^. Spectral tuning of both UGs is unlikely to be mediated through the β1 notch equivalent residue (Fig. 6a,b). Mutating helix-4 Phe equivalents in UG1 Y_777_F (or V; Fig. 6c) and UG2 L_622_F (or V; Fig. 6d) had almost no effect on the absorption spectra or U/G photocycles, except that dark states absorbed violet light (398 nm for Y_777_F in UG1; 389 nm L_622_V in UG2) instead of near-UV light (Supplementary Table S1). By contrast, mutation of β2 Phe equivalents F_684_ in UG1 and Y_537_ in UG2 had a large influence on spectral tuning of both dark and photoproduct states. The UG1 F_684_V variant failed to undergo a normal photoconversion (Fig. 6e). However, when the second point mutation was introduced by replacing helix-4 Tyr with either Phe or Val, the F_684_V Y_777_F (or V) double variant exhibited a strong violet (400 or 393 nm) and orange (591 or 583 nm) absorption in dark states and photoproducts (Supplementary Table S1), rather than the usual near-UV (384 nm) or green (560 nm) light-absorbing wild-type forms. By contrast, the UG2 Y_537_V single and Y_537_V L_622_F (or V) double mutants displayed red-shifted dark states and photoproducts (Fig. 6f). CD spectra of these double mutants also exhibited red-shifted peaks compared with 15*E* UGs (Supplementary Fig. S6), similar to that observed for the orange state of VO1^16^. These results indicate that the two UGs adopt different trapped-twist photoproduct conformations, mainly due to variation in residues corresponding to the Phe residues of AnPixJg2; helix-4 Phe has a clear influence on dark state conjugation, whilst both the dark state and photostate are directly influenced by the β2 Phe.

## Discussion

**Figure 7:**
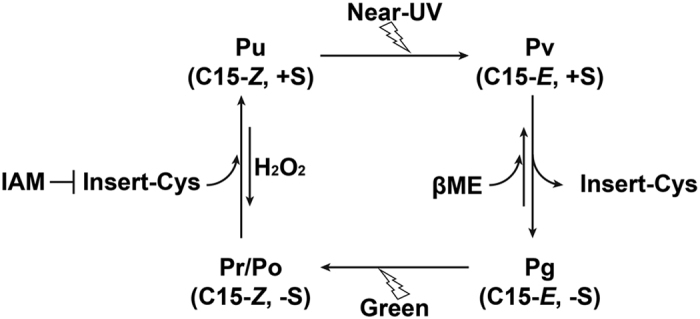
Proposed near-UV/green photocycle of Mbr3854g4 and Mbl3738g2. Near-UV and green photocycles involve light-dependent photoisomerisation of C15-*Z* PCB to the C15-*E* and light-independent reversible formation of the second thiol linkage at C10. The photo-labile second thiol linkage was probed using thiol reagents IAM, βME and peroxide. IAM and peroxide prevent the reformation of the second linkage. Peroxide also cleaves the second linkage in both 15*Z* and 15*E* photostates. βME is able to mimic the insert-Cys residue by forming the second thiol linkage to the chromophore C10 atom. Long black arrows indicate dark reactions that are favourable and hence occur rapidly. Short black arrows indicate dark reactions that are unfavourable and occur slowly. Pu, Pg, Pv, Pr and Po represent near-UV-, green-, violet-, red- and orange-absorbing species, respectively; +S and –S indicate thiol-ligated and thiol-free PCB, respectively.

In the present study, we obtained results that provide new insight into the photocycle of insert-Cys CBCRs, and identified several amino acid residues required for efficient photocycling and spectral tuning of both dark states and photoproducts. As shown in Fig. 7, photoconversion of near-UV-absorbing forms (Pu; 15*Z* PCB, thiol-ligated to insert-Cys) to green-absorbing forms (Pg; 15*E* PCB, insert-Cys thiol-free) leads to breakage of the second thioether linkage at C10^16^. In the 15*Z* Pu state, the insert-Cys residue is covalently attached to the bilin C10 atom, which effectively splits the conjugated system into two halves, with one half associated with the C- and D-rings, and the other with the A- and B-rings, allowing the C- and D-rings to absorb near-UV light. Exposure of 15*Z* Pu to near-UV light results in *Z*/*E* photoisomerisation of the C15 = C16 double bond between the C- and D-rings, which generates a violet-absorbing 15*E* intermediate photoproduct with the second Cys remaining intact (Pv; 15*E* PCB, insert-Cys-ligated). Subsequently, light-independent elimination of this second thiol linkage restores the chromophore π conjugation, which red-shifts the photoactive species to a green light-absorbing form (Pg). Reverse photoconversion of UG photoproducts occurs via formation of a further-red-shifted, red (UG1 Pr)- or orange (UG2 Po)-absorbing intermediate species (15*Z* PCB, insert-Cys-free). The thiol-based photocycles observed in the present study and the characteristic dynamic behaviour of the second thiol linkage are highly likely associated with structural changes in the insertion loop and the DXCF motif during the dark-to-light photoconversion^35^ since modification of these regions by deletion or substitution of selected residues significantly impaired photocycle activity.

Despite sharing similar general photocycle characteristics, a wide diversity in colour absorption in both dark states and photoproducts of insert-Cys-type CBCRs is achieved by spectral tuning. For instance, absorption peaks of dark states vary from near-UV (UG1 and 2, UB1) to violet (VO1). This variation also holds for photoproduct states that encompass blue (UB1), green (UG1 and 2) and orange (VO1) absorption maxima. This spectral diversity seems to be mediated by differences in the microenvironment of the PCB-binding pocket rather than changes in tertiary structure, especially within the immediate vicinity of the D-ring of the PCB chromophore, since the α-helical and β-sheet structure surrounding the D-ring of the chromophore in VO1 remains almost unchanged during the dark-to-light transition^35^. Consistent with this, 15*Z* and 15*E* forms of both UGs responded differently to the thiol modifying agent IAM (Fig. 3) and the thioether-destroying peroxide (Supplementary Fig. S7); although the dark state of UG1 was IAM accessible, this was inaccessible in UG2, and the photoproduct of UG1 was peroxide sensitive, whereas that of UG2 was peroxide insensitive. To date, there are no experimentally determined tertiary structures for insert-Cys CBCRs in publically available databases, making it difficult to correlate the differences in spectral tuning with structural information. Fortunately, some information can be gleaned from the structure of AnPixJg2 in its red-absorbing dark state^38^ that belongs to the same clade as insert-Cys CBCRs, according to phylogenetic analysis of CBCR GAFs^33^. Among the amino acid residues forming the PCB chromophore pocket, those corresponding to the β1 notch (I), β2 Phe (III), Phe in the DXCF motif (VII) and α4 Phe (X) residues (Fig. 1, Supplementary Fig. S1) sterically constrain the R/G cycles in AnPixJg2, NpR6012g4, NpR5113g2 and NpR3784 via the trapped-twist mechanism^30^,^34^. Among these four residues potentially responsible for the trapped-twisted geometry in insert-Cys CBCRs, replacement of Phe with Leu (VII) in all four insert-Cys CBCRs ruled out a possible role in colour tuning. By contrast, generation of singly or doubly substituted variants at position III alone, or in combination with substitution at helix-α4 (position X), resulted in species that were red-shifted in both dark (wild type = 384 nm *vs.* 393−400 nm in the variants) and light (wild type = 560 nm *vs.* 583−591 nm in the variants) photostates (Supplementary Table S1), suggesting that Phe equivalents at these positions are critical for spectral tuning for both photostates of UG1 and 2. Consistently, mutagenesis of these equivalents in NpR6012g4 resulted in variants with red-shifted photoproducts^30^.

The trapped-twist mechanism in the colour tuning of canonical red/green CBCRs predicts that chromophore constraint is the result of chromophore-protein interactions that determine the tilted geometry of the trapped chromophore. This model implies that a chromophore-binding pocket with a larger volume would constrain PCB conjugation only weakly, resulting in a species that absorbs at a longer wavelength than species with a tightly twisted geometry. To correlate amino acid residues equivalent to the β1 notch (I), β2 element (III) and α4 helix (X) with chromophore volume, *in silico* mutagenesis of these residues mimicking wild-type insert-Cys CBCRs and their variants was performed using the AnPixJg2 crystal structure in its dark state (PDB ID: 3W2Z) as template. The volume of the chromophore-binding pocket was then estimated as a proxy measuring changes in volume. Although only two or three of these residues were substituted to mimic respective insert-Cys CBCRs, the estimated volumes of the binding pockets of UG1, UG2 and UB1 were comparable, but significantly smaller than that of VO1 (Supplementary Table S2 and Fig. S8). Substitution of the β2 Phe with a smaller residue resulted in a substantial increase in the volume of the chromophore-binding pocket, whilst substitution of the β1 notch or helix α4 Phe residues had a much less pronounced effect. Interestingly, substitution of the helix α4 Phe had a significant impact on volume when the β2 Phe was replaced by a smaller residue, suggesting that the β2 residue is a key determinant of the volume of the chromophore-binding pocket. Overall, a larger chromophore pocket resulting from a smaller residue in the vicinity of the bilin D-ring leads to red-shifting of both dark and photoproduct states. Thus, in insert-Cys CBCRs, steric constraints in the chromophore pocket are likely determined by residues equivalent to the β2 Phe and helix α4 Phe. This *in silico* analysis should be corroborated in the future by determining crystal structures for wild-type and variant proteins.

In the present study, we demonstrated that insert-Cys CBCRs are able to sense green light, in addition to violet, blue and orange light, due to two spectral tuning mechanisms: a reversible second thioether linkage to the chromophore, and steric hindrance in the chromophore-binding pocket by Phe equivalents at the β2 and helix α4 positions. Among the four insert-Cys CBCRs investigated, UB1 was unique in terms of having a more stable second thioether linkage, and reversible formation of a second thioether link to PCB during photoconversion did not occur in this protein^16^. Further, among the three insert-Cys CBCRs with a labile second thioether linkage, the photoproducts of UG1 and 2 were blue-shifted relative to the orange absorption observed in the acid-denatured protein, but a comparable blue-shift was not observed in the VO1 photoproduct. Future structural studies could provide a more detailed understanding of the dynamic nature of the second thiol linkage, as well as the mechanism of spectral tuning. Upon completion of this work, insert-Cys CBCRs have now been characterised from heterocystous (*Nostoc punctiforme* ATCC29133) and nonheterocystous (*Microcoleus* B353) multicellular filamentous cyanobacteria. The high phylogenetic relatedness of these insert-Cys CBCRs to R/G single-Cys CBCRs such as AnPixJg2, NpR6012g4 and teal-DXCF CBCRs and the distinctness from the NpR3784 group^34^ suggest that the trapped-twist-dependent colour tuning adopted in UG CBCRs was derived from an ancestral R/G CBCR. Further studies are needed to investigate single-Cys CBCRs with DTY(/H)L and CH motifs as potential evolutionary intermediate sensors.

## Methods

### Strains and cultures

*Microcoleus* IPPAS B353 (*Microcoleus* B353), originally isolated from Lake Khilganta (Buryatiya, Russia; 50° 25′ N, 106° 53′ E, altitude: 606 m)^39^, was obtained from the Culture Collection of the Institute of Microbiology, Russian Academy of Sciences, Moscow, Russia. The strain was grown on S-solid media^40^ containing 2% agar or S-liquid media at 28 °C under continuous white light (30 μmol m^−2^ s^−1^)^17^.

### Expression and purification of recombinant His-tagged GAFs from PCB-producing *E. coli*

Plasmid pPL-PCB was used for PCB biosynthesis in *E. coli*^36^. GAF domains were amplified by PCR with appropriate primers (Supplementary Table S3) using *Microcoleus* B353 genomic DNA as template^17^. Appropriately restriction-digested PCR fragments were then cloned into the pBAD-MycHisC vector (Invitrogen, USA) to yield pBAD-GAF bacterial expression plasmids. *E. coli* LMG194 (Invitrogen) cells co-transformed with pBAD-GAF and pPL-PCB were grown overnight at 37 °C in 5 ml of rich media^36^ containing 50 μg/ml kanamycin and 200 μg/ml ampicillin. Recombinant GAF proteins were isolated and further purified as previously described^5^. Both photostates of recombinant GAFs were denatured by addition of a final concentration of 8 M urea/HCl (pH 2.0) at room temperature in the dark^5^,^37^.

### Spectrophotometric analyses

Steady-state absorbance was recorded at room temperature with a UV1601 spectrophotometer (Shimadzu, Japan). Photointerconversion of recombinant GAFs was triggered in the absorption cuvette using a UV-A tube (Model XX-40, Spectroline, 355 ± 28.6 nm) or halogen lamp (Osram LT05041) equipped with band-pass interference filters (Edmund Optics, 442 ± 10, 580 ± 10 and 671 ± 10 nm)^17^. Incident photon fluxes for forward and reverse photoconversions were 0.05 and 0.6 μmol m^−2^ s^−1^, respectively. Full photoconversion occurred within 3 min of illumination, and dark conversion was barely observed in any state. An electrically calibrated pyroelectric radiometer (ECPR, Model Rs5900, Laser Probe), which remained almost constant throughout the experiment (within 1%), was used to maintain a constant fluence rate throughout each experiment. CD spectra were acquired on a spectropolarimeter (Jasco J-815, USA) at room temperature and are presented as the smoothed average of three scans with buffer subtraction. Treatment of freshly prepared 3 mM IAM^16^ was performed for 3 min at room temperature. H_2_O_2_ treatment involved the addition of an equal volume of commercial 30% H_2_O_2_ for 2 min, followed immediately by spectroscopic analysis^16^. DTT and βME were added to a final concentration of 50 mM from freshly prepared 1 M stocks^16^,^22^.

### SDS-PAGE and in-gel zinc-dependent fluorescence assays

For zinc-dependent fluorescence assays^41^, purified CBCR GAF proteins were separated on a 12% (w/v) SDS-PAGE gel. Gels were then soaked in 20 mM zinc acetate at room temperature for 30 min in the dark, and imaged for fluorescence under UV-B excitation (302 nm) using a Bio-Rad Gel Doc 2000 equipped with a blue filter (480BP). Gels were stained with Coomassie Brilliant Blue R-250 (Bio-Rad).

### Bioinformatics

Multiple amino acid sequence alignments were carried out using MUSCLE^42^. For calculating the volume of the PCB-binding pocket, structures of UG1, UG2, VO1, UB1 and their variants were generated using the AnPixJ structure (PDB ID: 3W2Z). Volumes were calculated using the CASTp database (http://sts.bioe.uic.edu/castp/)^43^ and drawn using Chimera (https://www.cgl.ucsf.edu/chimera/)^44^.

### Note

In this study, we adopted nomenclature for CBCRs to represent proteins with single or multiple bilinbinding GAF domains. GAF domains are numbered from the N-terminus. Mbr3854 has four GAF domains, of which Mbr3854g1 is the most N-terminal. Throughout this article, the following colour code applies: near-UV (U), 300−395 nm; violet (V), 395−410 nm; blue (B), 410−480 nm; teal (T), 480−520 nm; green (G), 520−570 nm; yellow (Y), 570−580 nm; orange (O), 580−615 nm; red (R), 615−685 nm; far-red (FR), 685−750 nm.

## Additional Information

**How to cite this article:** Cho, S. M. *et al*. Hydrophobic Residues near the Bilin Chromophore-Binding Pocket Modulate Spectral Tuning of Insert-Cys Subfamily Cyanobacteriochromes. *Sci. Rep.* **7**, 40576; doi: 10.1038/srep40576 (2017).

**Publisher's note:** Springer Nature remains neutral with regard to jurisdictional claims in published maps and institutional affiliations.

## Supplementary Material

Supplementary Information
