# Supplementary material for: Hydrophobic Residues near the Bilin Chromophore-Binding Pocket Modulate Spectral Tuning of Insert-Cys Subfamily Cyanobacteriochromes
Source: Sci Rep. 2017 Jan 17;7:40576. doi: 10.1038/srep40576 (PMC5240096; doi:10.1038/srep40576)
Supplement: Supplementary Information [file srep40576-s1.docx]

**Supplementary materials**

**Hydrophobic Residues near the Bilin Chromophore-Binding Pocket Modulate Spectral Tuning of Insert-Cys Subfamily Cyanobacteriochromes**

Sung Mi Cho^1,2,+^, Sae Chae Jeoung^3,+^, Ji-Young Song^1^, Ji-Joon Song^4^, and Youn-Il Park^1^

^1^Department of Biological Sciences, Chungnam National University, Daejeon 34134 Korea

^2^Unit of Polar Genomics, Korea Polar Research Institute, Incheon 21990 Korea (Present affiliation)

^3^Center for Advanced Measurement and Instrumentation, Korea Research Institute of Standards and Science, Daejeon 34113 Korea

^4^Department of Biological Sciences, Korea Advanced Institute of Science and Technology, Daejeon 34141 Korea

^+^These authors contributed equally to this work

^*^Corresponding author: Youn-Il Park, Email: yipark@cnu.ac.kr; Phone/Fax numbers: +82-42-8215493/+82-42-8229690

**Supplementary Table S1**. Photochromic properties of wild type and variant forms of UG1 Mbr3854g4 and UG2 Mbl3738g2.

| Protein | λ_max_ (15*Z*;15*E*)^a^ | %ΔΔA^b^ | Protein | λ_max_  (15*Z*;15*E*) | %ΔΔA |
| --- | --- | --- | --- | --- | --- |
| UG1  wild type | 384; 560 | 100 | UG2  wild type | 381; 556 | 100 |
| C_672_A | 387; 558 | 100 | L_525_A | - ^c^ | - |
| F_684_V | 413; - | - | Y_537_V | 400; 592 | 29 |
| C_713_A | 620; - | - | C_569_A | 592; - | - |
| D_739_A | 396; 579 | 11 | D_583_A | - | - |
| D_739_E | 393; 563 | 22 | D_583_E | 399; 566 | 21 |
| Y_741_F | 384; 560 | 91 | Y_585_F | 388; 554 | 71 |
| Y_741_C | 389; 568 | 12 | Y_585_C | 399; 566 | 24 |
| C_713_A Y_741_C L_742_F | 392; 582 | 5 | C_569_A Y_585_C L_586_F | 410; 529 | 7 |
| E_744_D | 384; 560 | 95 | E_588_D | 381; 556 | 70 |
| E_744_H | 384; 560 | 95 | E_588_H | 381; 556 | 70 |
| Y_777_F | 398; 559 | 75 | L_622_F | 389; 559 | 68 |
| Y_777_V | 398; 561 | 83 | L_622_V | 389; 556 | 68 |
| F_684_V Y_777_F | 400; 591 | 28 | Y_537_V L_622_F | 399; 590 | 23 |
| F_684_V Y_777_V | 393; 583 | 24 | Y_537_V L_622_V | 399; 583 | 31 |
| ΔI^d^ Y_741_C | 425; 591 | 6 |  |  |  |

^a^ Native peak wavelengths were estimated by fitting of absorption spectra. The longest wavelength (nm) in each photostate is presented as 15*Z*; 15*E* pairs.

^b^ %ΔΔA, percent photoconversion efficiency was determined by subtracting the Pg spectrum from the Pu spectrum, subtracting the resulting difference minimum from the difference maximum, and normalizing to the wild type spectrum that was designated 100%^1^.

^c^ -, not detected due to failure of recombinant variant proteins in either chromophorylation or photoconversion.

^d^ I, amino acids in the insert region (residues 699−730) were deleted.

**Supplementary Table S2. Consensus residues in the CBCR lineage and chromophore-binding pocket size.**

| CBCR | Variant | β1 | β2 | DXCF/CH-motif | Helix | β6 | Volume (Å^3^)^a^ | Peak (nm)^b^ |
| --- | --- | --- | --- | --- | --- | --- | --- | --- |
| Red/green (R/G) | |  |  |  |  |  |  |  |
| AnPixJg2 | Wild type | Ala | Phe | DXXL/CH | Phe | Tyr | 663.4 | 648 |
| Insert-Cys | |  |  |  |  |  |  |  |
| Mbr3854g4 (UG1) | Wild type | Cys | Phe | DTYL/CY | Tyr | Tyr | 662.8 | 384 |
|  | F_684_V |  | Val |  | Tyr |  | 849.3 | 400 |
|  | Y_777_F |  | Phe |  | Phe |  | 664.0 | 398 |
|  | Y_777_V |  | Phe |  | Val |  | 664.0 | 395 |
|  | F_684_V Y_777_F |  | Val |  | Phe |  | 849.6 | 400 |
|  | F_684_V Y_777_V |  | Val |  | Val |  | 968.9 | 393 |
| Mbl3738g2 (UG2) | Wild type | Leu | Tyr | DTYL/CY | Leu | Tyr | 668.3 | 381 |
|  | Y_537_V |  | Val |  | Leu |  | 845.2 | 405 |
|  | L_622_F |  | Tyr |  | Phe |  | 668.5 | 389 |
|  | L_622_V |  | Tyr |  | Val |  | 668.5 | 389 |
|  | Y_537_V L_622_F |  | Val |  | Phe |  | 776.0 | 399 |
|  | Y_537_V L_622_V |  | Val |  | Val |  | 910.5 | 399 |
| Npr1597g2 (UB1) | Wild type | Ala | Phe | DTYL/CY | Tyr | Tyr | 663.4 | 378 |
| NpF2164g3 (VO1) | Wild type | Val | Val | DTYL/CY | Phe | Tyr | 827.0 | 398 |
| DXCF (B/G) | |  |  |  |  |  |  |  |
| TePixJ | Wild type | Ile | Val | DXCF/CH | Leu | His |  |  |
|  | 4GLQ |  |  |  |  |  | 728.4 | 433 |
|  | 3VV4 |  |  |  |  |  | 930.8 | 531 |

^a^ Chromophore pocket volumes of the dark state of AnPixJg2 and its site-directed mutagenesis variants mimicking wild-type insert-Cys CBCRs, as well as single, double or triple mutants of UGs were estimated using the CASTp database (http://sts.bioe.uic.edu/castp/). The AnPixJg2 crystal structure in its Pr state (PDB ID: 3W2Z) was used for *in silico* site-directed mutagenesis of amino acid residues at the β1 notch (I), β2 Phe (III) and α4 helix Phe (X) positions before estimation of pocket volume. 4GLQ and 3VV4 were used for calculation of dark and photoproduct volumes of TePixJ, respectively.

^b^ Absorption peaks for the dark state of AnPixJg2 and wild-type and variant UGs are given. For TePixJ, absorption peaks for both dark (4GLQ) and photoproduct (3VV4) states are provided.

**Supplementary Table S3.** Primers used in this study.

| Primer | Direction | Sequence |
| --- | --- | --- |
| **Mbr3854g4 UG1 variants** | |  |
| Y672A | F | GTC TGT GTC GCT CAA TTC G |
|  | R | CGA ATT GAG CGA CAC AGA C |
| F684V | F | GAG TGG GTC CGT TAT TGC |
|  | R | GCA ATA ACG GAC CCA CTC |
| C713A | F | CAG CCA AGC AGA CAA CAT |
|  | R | ATG TTG TCT GCT TGG CTG |
| ΔI | F | CAA GCC AGC GGC CCC |
|  | R | GGG GCC GCT GGC TTG CTG CAC CAG GGG GGC |
| D739A | F | CCA GTC GGG CTA CCT ATC TG |
|  | R | CAG ATA GGT AGC CCG ACT GG |
| D739E | F | CCA GTC GGG AAA CCT ATC TG |
|  | R | CAG ATA GGT TTC CCG ACT GG |
| Y741C | F | GGA TAC CTG TCT GCA AGA G |
|  | R | CTC TTG CAG ACA GGT ATC C |
| Y741F | F | GAT ACC TTT CTG CAA GAG AC |
|  | R | GTC TCT TGC AGA AAG GTA TC |
| Y741C/L742F | F | GGA TAC CTG TTT TCA AGA GAC |
|  | R | GTC TCT TGA AAA CAG GTA TCC |
| E744D | F | CGT GTC TTG CAG ATA GGT ATC |
|  | R | GAT ACC TAT CTG CAA GAC ACG |
| E744H | F | CGT ATG TTG CAG ATA GGT ATC |
|  | R | GAT ACC TAT CTG CAA CAT ACG |
| Y777F | F | CGA ACA ATT T CA ATG TCG C |
|  | R | GCG ACA TTG AAA TTG TTC G |
| Y777V | F | CGA ACA AGT TCA ATG TCG C |
|  | R | GCG ACA TTG AAC TTG TTC G |
| **Mbl3738g2 UG2 variants** | |  |
| L525A | F | CGC GTT GCC GTC TAC CGC TTT |
|  | R | AAA GCG GTA GAC GGC AAC GCG |
| Y537V | F | TGG AAC CGT TGT CGC TGA A |
|  | R | TTC AGC GAC AAC GGT TCC A |
| C569A | F | GCA ACC AAA CGA CTC GAT GCC AGT |
|  | R | CAC TGG CAT CGA GTC GTT TGG TT |
| D583A | F | TAG TTC AAG CCA CCT ATC TAC AA |
|  | R | TTG TAG ATA GGT GGC TTG AAC TA |
| D583E | F | TAG TTC AAG AGA CCT ATC TAC AA |
|  | R | TTG TAG ATA GGT CTC TTG AAC TA |
| Y585C | F | GTT CAA GAT ACC TGC CTA CAA GA |
|  | R | TCT TGT AGG CAG GTA TCT TGA AC |
| Y585F | F | TCA AGA ATC CTT TCT ACA AGA |
|  | R | TCT TGT AGA AAG GTA TCT TGA |
| Y585C/L586F | F | CAA GAT ACC TGC TTC CAA GAA |
|  | R | TTC TTG GAA GCA GGT ATC TTG |
| E588D | F | CCA TAA TTG TCT TGT AGA TAG GTA TC |
|  | R | GAT ACC TAT CTA CAA GAC AAT TAT GG |
| E588H | F | CCA TAA TTG TGT TGT AGA TAG GTA TC |
|  | R | GAT ACC TAT CTA CAA CAC AAT TAT GG |
| L622F | F | TTA GAA CAA TTC CAG GCT CGT |
|  | R | ACG AGC CTG GAA TTG TTC TAA |
| L622V | F | TTA GAA CAA GTT CAG GCT CGT |
|  | R | ACG AGC CTG AAC TTG TTC TAA |


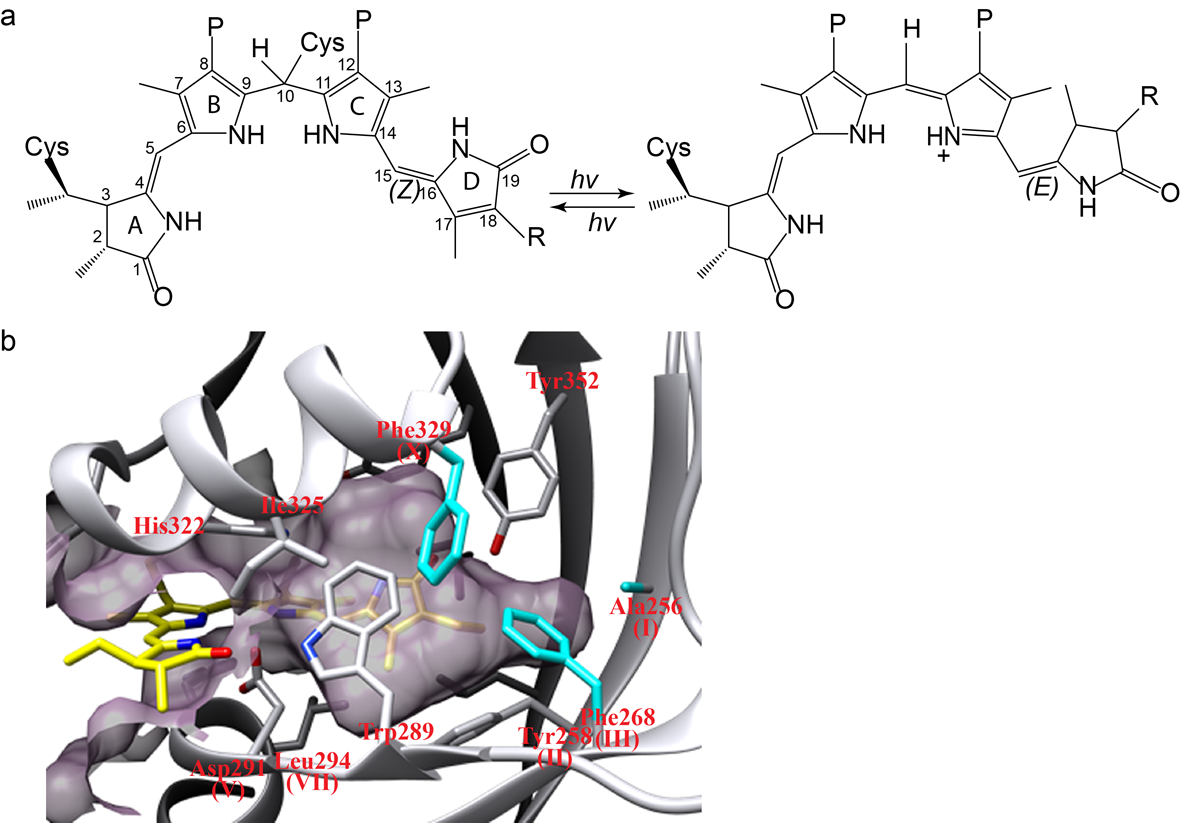


**Supplementary Figure S1**. **Reversible splitting of π-conjugation in the PCB chromophore via thioether linkage with the second Cys residue and the chromophore-binding pocket of AnPixJg2 GAF.** (**a**) Reversible formation of the second thioether linkage to the phycocyanobilin (PCB) chromophore. In Phys and CBCRs, the canonical first Cys residues bind to the linear tetrapyrrole chromophore at the 3’-position of the A-ring ethylidene group. In dual-Cys CBCRs that have a second Cys within the DXCF motif or within the insert region, the second thioether linkage occurs at the C10 position of the bilin chromophore. PCB adopts C5-*Z*, *syn*/C10-*Z*, *syn*/C15-*Z*, *anti* configurations in the parental dark state. A Z/E isomerization of the C15=C16 double bond between rings C and D occurs during photoconversion. P, propionate; R, ethyl. (**b**) The chromophore-binding pocket of the AnPixJg2 GAF domain with PCB in the red-absorbing Pr state. β2 Phe and the helix α4 Phe, together with β1 notch Ala and DXCF motif Leu, form a pocket that accommodates the PCB chromophore, as shown in the AnPixJg2 structure (PDB ID: 3W2Z). The chromophore-binding pocket is drawn in surface representation (light purple). Labels refer to amino acid sequence numbers, and PCB is colored yellow and shown in stick form. Key residues denoted in the multiple sequence alignment in Fig. 1 and discussed in the main text are indicated with Roman numerals.

**
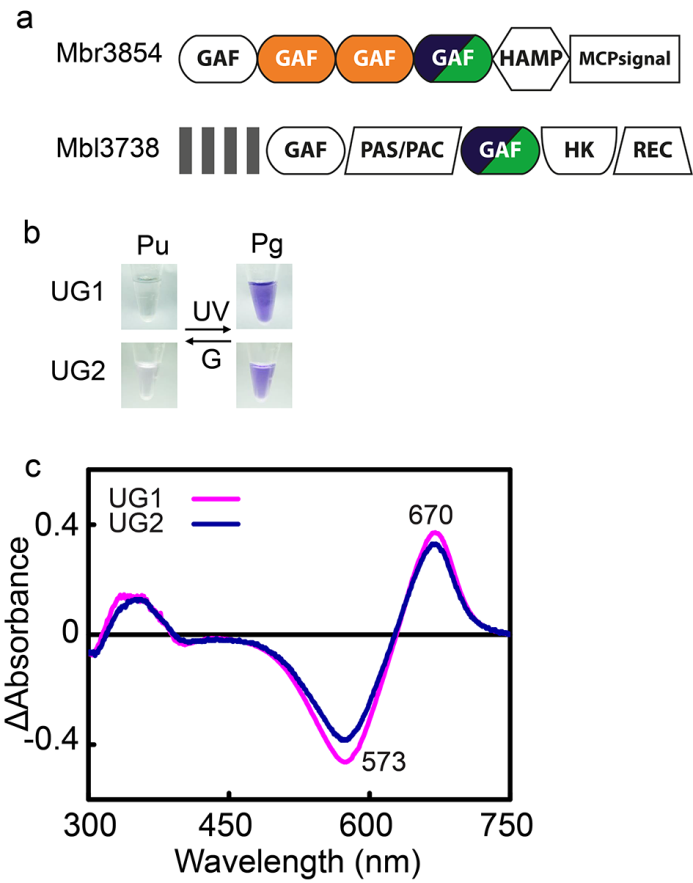
**

**Supplementary Figure S2**. **Newly identified insert-Cys CBCRs Mbr3854g4 (UG1) and Mbl3738g2 (UG2) from *Microcoleus* B353.** (**a**) Domain architecture of Mbr3854 and Mbl3738. Color-coded GAF domains represent photocycles in dark and light states. HK, histidine kinase with ATPase; REC, receiver domain; HAMP, histidine kinase, adenylyl cyclases, methyl-accepting proteins, and phosphatases; MCP, methyl-accepting chemotaxis protein; PAS, period circadian protein, aryl hydrocarbon receptor nuclear translocator, and single-minded homolog 1. (**b**) Color changes of recombinant GAF proteins UG1 and UG2 in respect to their near-UV (Pu) and green (Pg) light-absorbing species. (**c**) Difference spectra of wild type UG1 and UG2.


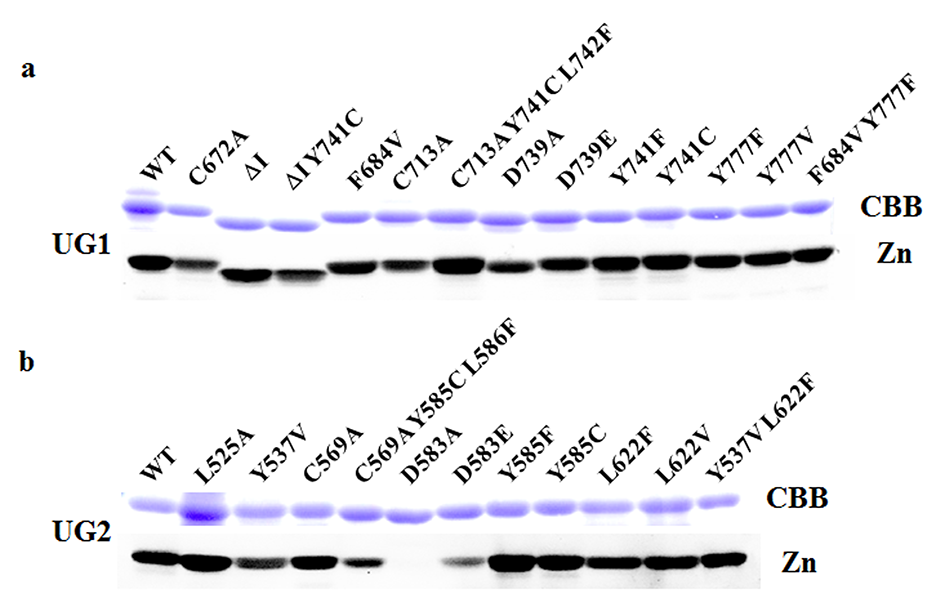


**Supplementary Figure S3. Zinc blot and CBB staining of His-tagged UG1-GAF (A) and UG2-GAF (B) wild type and variant proteins purified from PCB-producing *E. coli*.** SDS-PAGE gels are shown after staining with Coomassie Brilliant Blue (CBB), and fluorescence is shown after soaking the gel in Zn^2+^ solution (Zn).

**
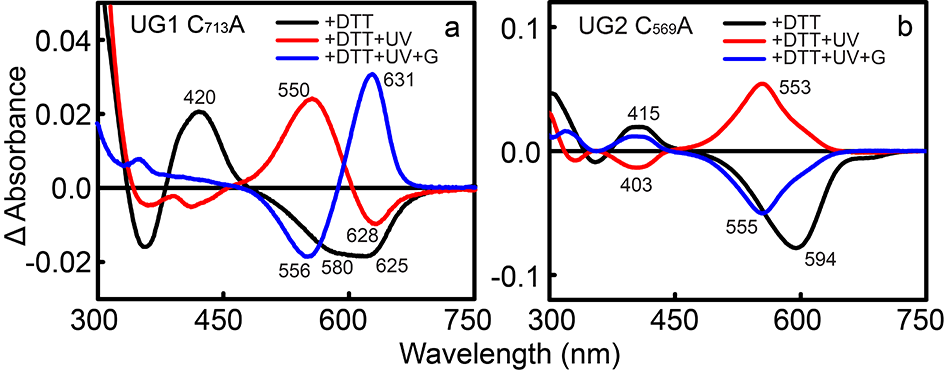
**

**Supplementary Figure S4. Difference spectra show that DTT treatment restores the photocycle of insert-Cys mutants UG1 C_713_A (a) and UG2 C_569_A (b).** Both mutants were treated with 50 mM DTT (red) in the ground state, irradiated with near-UV light (blue), then illuminated with green light (pink).


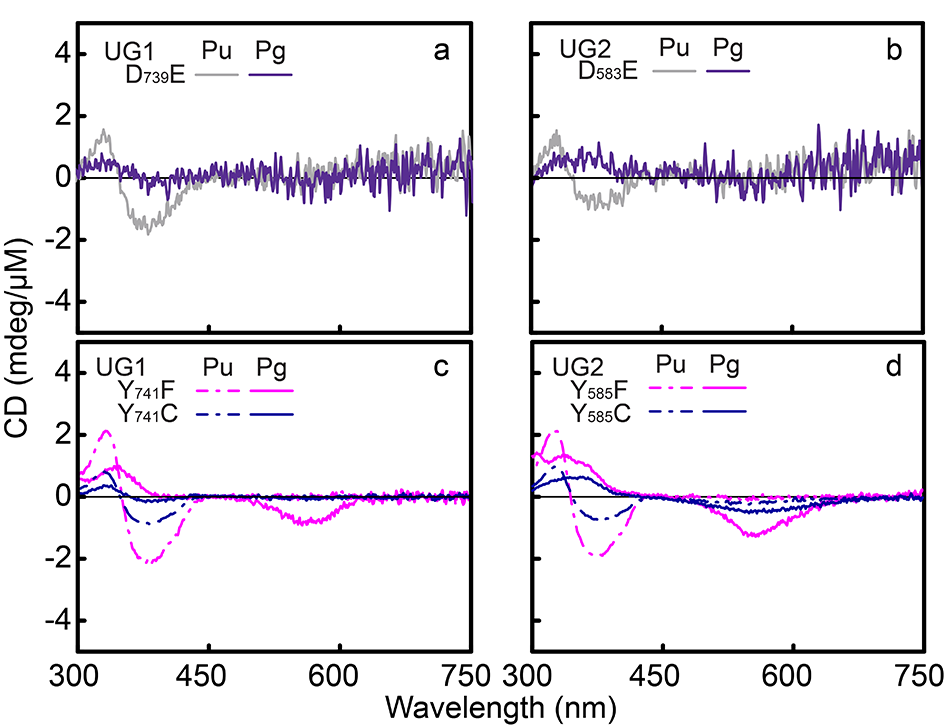


**Supplementary Figure S5. DXCF motif of insert-Cys CBCRs are required for photoisomerization.** CD spectra of Asp variants of UG1 (D_739_A and D_739_E) (**a**) and UG2 (D_583_A and D_583_E), and Tyr variants of UG1 (Y_741_F and Y_741_C) (**b**) and UG2 (Y_585_F and Y_585_C) (**c**). Spectra were recorded for the dark state, or after near-UV light irradiation.


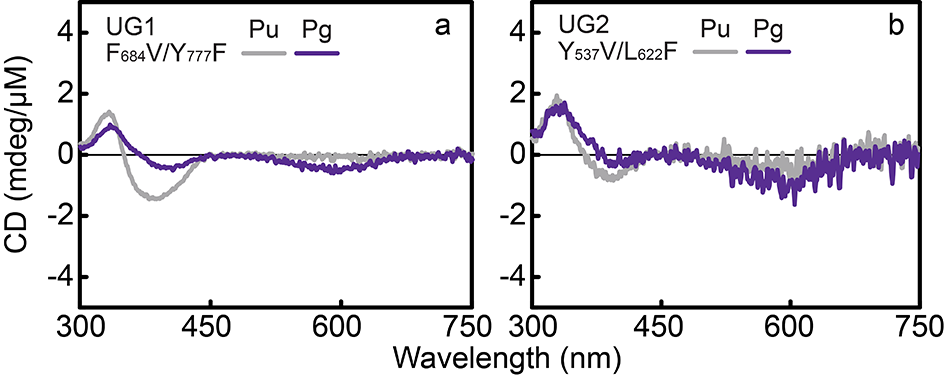


**Supplementary Figure S6. Hydrophobic residues in helix α4 and β2 elements govern spectral tuning in the dark state and in photoproducts.** CD spectra of β2 and helix Phe double F_684_V Y_777_F in UG1 (**a**) and Y_537_V L_622_F in UG2 (**b**).

**
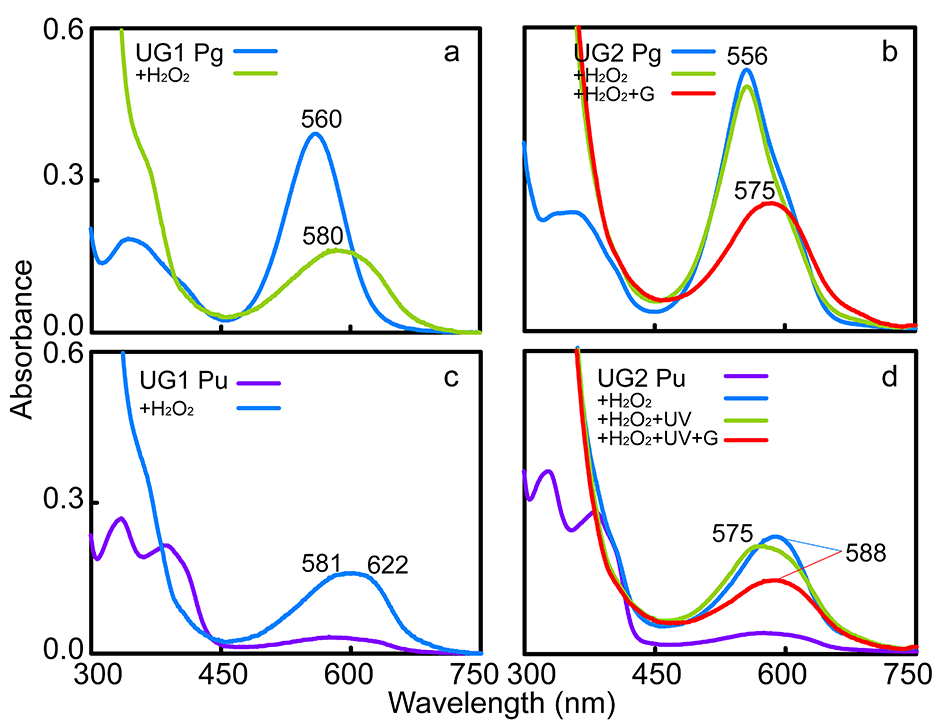
**

**Supplementary Figure S7. Differential susceptibility of the second thioether linkage of UG1 and UG2 to H_2_O_2_ treatment.** Reverse photoconversion (15*E* to 15*Z*) (**a, c**) and forward photoconversion (15*Z* to 15*E*) (**b, d**) for UG1 (**a, b**) and UG2 (**c, d**) were examined after treatment with H_2_O_2_. Photoproteins were converted to the 15*E* state (blue), followed by reaction with H_2_O_2_ in darkness to yield 15*E* chemical products (green). The 15*E* chemical products were then illuminated when appropriate to generate 15*Z* photoproducts (red). The UG1 dark state is sensitive to H_2_O_2_ treatment, whilst that of UG2 is resistant, and this is also true of the photostates.

**
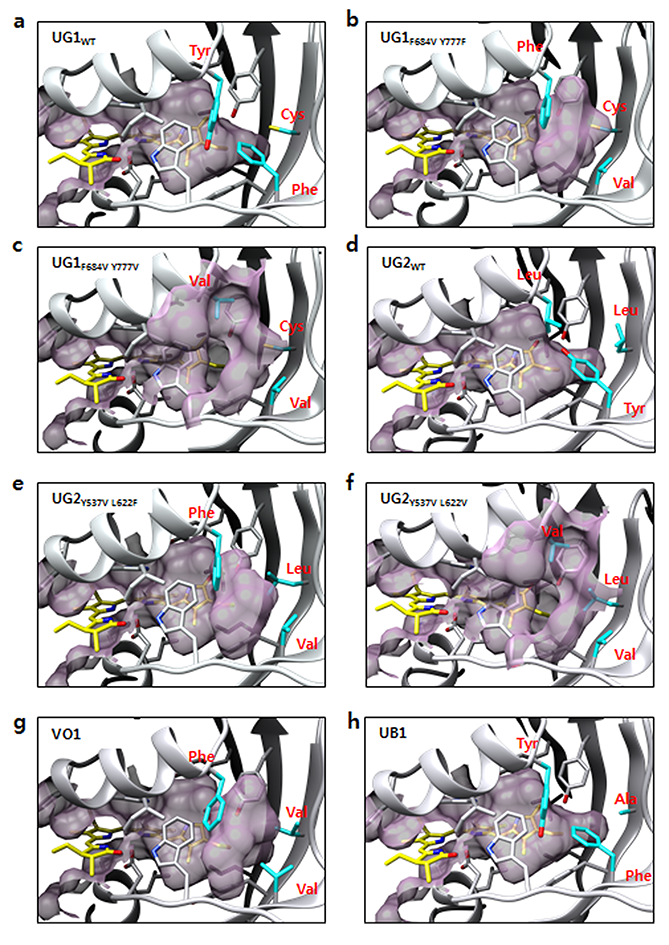
**

**Supplementary Figure S8. Variations in amino acids alter the size of the PCB chromophore-binding pocket.** The chromophore-binding pockets mimicking wild-type insert-Cys CBCRs of UG1, UG2, VO1, UB1, and UGs variants are drawn in surface representation (light purple). The PCB chromophore is shown in yellow, and amino acids forming the binding pocket are in stick representation. Amino acid variations at the β1 notch, β2 Phe, and α4 positions are colored light blue. (**a**) UG1 wild type (WT); (**b**) UG1 F_684_V Y_777_F; (**c**) UG1 F_684_V Y_777_F; (**d**) UG2 wild type; (**e**) UG2 Y_537_V L_622_F; (**f**) UG2 Y_537_V L_622_V; (**g**) VO1; (**h**) UB1.

**References**

1. Fischer, A. J. *et al.* Multiple roles of a conserved GAF domain tyrosine residue in cyanobacterial and plant phytochromes. *Biochemistry*. **46**, 15203-15215 (2005).
